# Rapid assessment of home management of malaria among caregivers in parts of south eastern Nigeria

**Published:** 2011-11-02

**Authors:** Uche Chukwuocha

**Affiliations:** 1 Department of Public Health Technology, Federal University of Technology, Owerri, Nigeria

**Keywords:** Malaria, assessment management, caregivers, Nigeria

## Abstract

**Background:**

This study was carried out to rapidly access the practice of home management of malaria by caregivers and community health workers in a rural part of South Eastern Nigeria between March and October, 2010.

**Methods:**

Structured, pretested questionnaires, focus group discussions and oral interviews were used to collect data from 300 consenting individuals.

**Results:**

Most of the participants/respondents were civil servants/teachers (44.3%). About 88.3% of them recognized malaria as an illness, 81.0% perceived it was transmitted by mosquito bites. Malaria diagnosis at home was mainly by noticing fever, headache, cough, and pains (86.0%). Most primary action was sought by going to hospitals/health centers (62.3%) and choroquine (46.7%) was the preferred antimalarial drug. Some of the factors hindering effective home management of malaria in the area included ignorance (13.0%); use of fake drugs (50%) and wrong diagnosis (19.1%).

**Conclusion:**

This study shows that there is some awareness about malaria and its management in the study area. There is however need to improve and sustain the strategy, placing more emphasis on educating the people on current drug protocols to achieve better results in controlling and combating malaria especially at the local levels.

## Background

Malaria is a life threatening parasitic disease transmitted through the bite of infected female anopheles mosquitoes. It remains a major public health problem in Nigeria being the most common cause of hospital attendance in all age groups in all parts of the country [1].

In Nigeria 98% of all cases of malaria is due to *Plasmodium falciparum* and this is the specie that is responsible for the severe form of the disease that leads to death [2].

According to Chukwuocha and et al [3], malaria is among the common causes of childhood mortality in the country. It is estimated that 50% of the population has at least one episode of malaria each year while children under 5 have an average of 2-4 attacks in a year. Malaria has serious and severe negative effects on maternal health and birth outcomes, it causes maternal anaemia, low birth weight and increases miscarriages [4].

The disease not only imposes a heavy cost on the country's income but also retards its rate of economic growth and invariably its level of economic development. Steketee et al [5] had previously observed that the disease limits the productivity of a country's two major assets, its people and its land. The cost of prevention and treatment consumes scarce household resources and the burden on the public health sector is imputed on the allocation of government resources [6].

Despite efforts to provide effective antimalarial drugs and other interventions for its prevention and treatment, malaria has remained a lingering public health problem. Some local communities face considerable difficulty while getting access to these services due to many barriers hence most malaria victims die because of lack of health care close to their homes or because their conditions are not diagnosed by qualified trained health workers or caregivers as the case may be.

Therefore, for effective control, home management of malaria has become one of the important strategies to reach the precious target of malaria elimination especially in remote areas [7]. However for the effective implementation of the home management of malaria initiative to be achieved, caregivers and community health workers should be able to recognize the illness early, obtain and use correct drugs appropriately; especially in children under 5 (five) years of age and be able to refer immediately to the hospital or health center if treatment does not work after three days. It is very important that caregivers learn to recognize the malaria symptoms early particularly fever.

However the implementation and practice of this, all important, community based and cost effective strategy has faced bottlenecks in several endemic areas [2,7]. It is therefore very important that factors responsible for these setbacks should be identified and corrected so that the strategy can be effectively implemented to achieve more sustainable goals.

This study is aimed at rapidly assessing home management of malaria practice among care givers and community health workers identify those factors that hinder effective practice and proffer solutions using Umunneochi Local Government Area of Abia State, South Eastern Nigeria as a case study.

## Methods

### Study area

The study area was Umunneochi Local Government Area of Abia State, South Eastern Nigeria located between longitude 6.2°–6.4° north and latitude 7.3° – 7.5° east.

It has an estimated population of 163, 199 people according to the 2006 census figure [8]. The inhabitants are mostly subsistence farmers. Some inhabitants also engage in retail and petty trading. Umunneochi shares the same climatic conditions with the rest of South-Eastern Nigeria, a tropical region dominated by two seasonal conditions. The hot dry season starts from November and lasts till April; it alternates with the rainy season which lasts from May to October. The location of Umunneochi and its climatic/environmental conditions favour the breeding of infectious mosquitoes hence the inhabitants suffer frequent episodes of malaria.

### Sample and sampling technique

A total number of 300 consenting individuals were randomly selected for this study from different churches on different Sundays through balloting. This was to ensure that different categories of people were represented. The samples were drawn from mothers in the three zones of the Local Government Area, caregivers and community health workers, nurses and health officers.

### Instrument for data collection

Four (4) instruments were used for data collection, that is: Structured, pretested questionnaires, personal observations, oral interviews and focus group discussions. Questionnaires were administered to the respondents in English and Igbo Languages after Church services by trained field assistants. Community health workers, mothers, nurses and health officers were also interviewed. The only health center located in the Local Government Area was visited during maternal and child health (MCH) days and observations were made.

### Validation and reliability of the instruments

Special visits were made to the health center and Local Government Health Department during maternal and child health days/immunization days. Mothers who brought their children for the immunization exercise were interviewed. In the process of observation, some staff of the health center and health department who were involved in the immunization programme also granted interviews. The objective of this was to give credibility to the responses from the community, to what was observed and to the questions and responses from respondents in the questionnaire.

### Administration of instruments

The questionnaire was given to and retrieved from the respondents by the researcher personally. This action was to prevent the preven loss of questionnaires.

### Data analysis

Data was tested using chi-square (x^2^). This is the most common non-parametric test developed by Karl Pearson in 1900 [9]. It is appropriate for both nominal and ordinary levels of data. The purpose of chi-square test is to determine how well an observed set of data fits an expected test.

## Results

### Occupational distribution of respondents in the study area

Table 1 shows the occupational distribution of respondents involved in the study. About 18 (6.0%) were students, 133 (44.3%) were civil servant/teachers, 52 (17.3%) were farmers/traders, 41 (13.7%) were housewives, 38 (12.7%) were healthcare professionals (Nurse, Lab Technologists), 9 (3.0%) were fishermen, 3 (1.0%) were business men and women while 6 (2.0%) represented other occupational groups.

**Table 1:** Occupational distribution of the respondents in the study area

Occupation	Frequency	Percentage
Student	18	6.0%
Civil servant (teacher)	133	44.3%
Farmer/trader	52	17.3%
Housewife	41	13.7%
Professionals (Nurse, Lab technologist) etc	38	12.7%
Fishermen	9	3.0%
Business men/women	3	1.0%
Others	6	2.0%
Total	300	100%

(X^2^= 4.76, df=1, P<0.95)

### Perception about the concept of malaria

About 88.3% had the right perception that malaria is an illness/disease, 8.7% believed that malaria is a spiritual problem while 3.0% perceived that malaria is a result of nutritional imbalance (Table 2).

**Table 2:** Perception about the concept of malaria in the study area

Causes of malaria	Frequency	Percentage
An illness/disease	265	88.3%
A spiritual problem	26	8.7%
Nutritional imbalance	9	3.0%
Total	300	100%

(X^2^= 2.31, df=1, P<0.95)

### Responses on recognition/ home diagnosis of malaria in children

Table 3 depicts respondents’ recognition/ diagnoses of malaria at home in their children. About 86.0% recognized malaria by constant fever, headache, cough and pains, 10.0% recognized the disease by frequent cold, catarrh and loss of appetite and convulsion. While 4.0% recognized the disease through weakness of the body and vomiting.

**Table 3:** Responses on home diagnosis of malaria in children

Symptoms	Frequency	Percentage
Constant fever, headache, cough and pains	258	86.0%
Frequent cold, catarrh and loss of appetite and convulsion	30	10.0%
Weakness of the body and vomiting	12	4.0%
Total	300	100%

(X^2^= 5.86, df=1, P<0.95)

### Primary action sought when symptoms are noticed

About 28% of respondents reported taking medicine bought from a chemist shop, 62.2% reported going to hospital/health centers for treatment, 8.7% reported going to a herbal doctor for treatment while 1.0% admitted going to church for prayers(Table 4).

**Table 4:** Primary action sought when symptoms are noticed

Symptoms	Frequency	Percentage
Take medicine bought from chemist	84	28.0%
Go to health centre/hospital	187	62.3%
Go to church for prayers	3	1.0%
Go to herbal doctor	26	8.7%
Total	300	100%

(X^2^= 3.42, df=1, P<0.95)

### Pattern of antimalaria drug treatment

Table 5 summarizes the pattern of anti malaria drug treatment. About 46.7% used chloroquine, 12 (4.0%) used sulfadoxine-pyrimethamine brands, 121 (40.3%) used Athemisin based Combination Therapies (ACTs) while 27 (9.0%) used quinine.

**Table 5:** Pattern of anti-malaria drug treatment

Anti-malaria drug	Frequency	Percentage
Chloroquine	140	46.7%
Sulfadoxine	12	4.0%
ACT	121	40.3%
Quinine	27	9.0%
Total	300	100%

(X^2^= 3.86, df=1, P<0.95); ACT: Artemisinin based combination therapy

### Factors hindering proper home management of malaria

About 13.0% noted illiteracy/ignorance as a factor that hindered proper malaria home management. Majority of respondents (50.0%) perceived that use of fake drugs and lack of money/drug resistance was a hindering factor. However, 19.1% reported improper use of insecticide treated nets and wrong diagnosis as factors while 17.0% agreed that an unclean environment, under and over dosage of drugs were factors that hindered proper home management of malaria (Table 6).

**Table 6:** Factors hindering proper home management of malaria

Factors	Frequency	Percentage
Illiteracy/Ignorance	34	13.0%
Fake drugs and lack of money/drug resistance	131	50.0%
Improper use of insecticide treated net/wrong diagnosis	50	19.1%
Unclean environment, under and over dosage of drugs	47	17.9%
Total	262	100.0%

(X^2^= 5.28, df=1, P<0.95)

## Discussion

In this study, it was observed that care-givers of children reporting malaria illness were knowledgeable about the disease and common symptoms associated with it. This is expected from a population with majority of people being exposed to public health issues as an occupational hazard. The respondents included civil servants and teachers and other professionals such as, nurses, laboratory technologist with farmers as well as students and housewives. This indicates the influence of occupation on knowledge of infectious diseases as reported by previous studies [1,9].

Most respondents in this study had the right perception about the concept of malaria; as an illness or disease in contrast to previous beliefs in certain areas that malaria was a spiritual problem or a manifestation of nutritional imbalance [10]. This may be due to frequent exposure to health issues and enlightenment about malaria.

The study also found a widely held perception of mosquito bite as the cause of malaria. Although other causes such as, eating too much oil, were reported. This perception of the right cause of malaria contradicts findings in other parts of South Eastern Nigeria [11] which reported perceptions of; heat from sun light, fried food and hard work, as causes of malaria. Public health education by health officers may have brought about the right perception of malaria causes in the study area.

Diagnosis of malaria at home by care-givers was mostly done by recognizing symptoms such as, constant fever, headache, cough and pains, loss of appetite etc. This concurs with a previous report in the Imo River Basin of Nigeria [7] and is in line with methods of effective management of malaria at home [12]. This involves early recognition of malaria symptoms followed by prompt treatment.

An improvement on previous reports on primary actions sought for malaria illness was recorded in the present study. A high number of respondents reported seeking treatment at the hospitals and health centers when symptoms were noticed. Previously, reasons such as non- availability of doctors, distance from home and long waiting periods for not attending health facilities were given [11]. However the efforts of government in public health delivery and increased awareness on the people may have contributed to this positive development.

Antimalaria drug treatment pattern shows that although chloroquine is still mostly used for malaria treatment, utilization of ACTs was also high. This indicates that the respondents are adopting the current anti malaria drug policies and are probably aware of parasite resistance to the drugs previously used. This result contradicts previous report [8] on utilization of sulphadoxine -pyrimethamine as a first line drug for malaria treatment in parts of Nigeria.

A high number of respondents in this study perceived that adequate knowledge of home management of malaria among mothers and care-givers will go a long way towards helping to solve the problem of malaria. They agreed that part of the problems faced in malaria control is ignorance among the people on ways of managing the illness. It has also previously been noted that public education on malaria would be veritable in arming the people effectively for malaria control [13].

Majority of the respondents attributed use of fake drugs and lack of money as well as drug resistance as factors hindering proper home management of malaria. Other factors reported include illiteracy and wrong diagnosis. Previous studies [14–16] also reported similar hindering factors to proper management of malaria. This calls for concerted action against fake and substandard drugs and also empowering the people so that they can make the right choices to achieve effective health care.

## Conclusion

Malaria has remained a major public health problem in Nigeria despite concerted efforts to provide effective measures for its control and management. The disease is the most common cause of hospital attendance in Nigeria and most people in rural communities suffer due to lack of health care close to their homes. Many malaria victims die because of not getting timely access to these services due to many barriers. Home management of malaria has therefore become a veritable and important strategy in the management and control of malaria. Malaria can actually be managed effectively at home by care-givers and mothers. Effective home management of malaria will lead to significant reduction in malaria mortality and morbidity. Moreover, managing malaria at home improves maternal health positively hence a reduction in miscarriages, low birth weight, and reduction in maternal anemia [7]. Equally, findings show that adequate education of the people on effective home management of malaria will contribute significantly in solving the malaria problem [11].

However, certain factors such as fake drugs, wrong diagnosis, drug resistance, poverty etc have been shown to militate against the effective implementation and sustainability of the strategy. Political will from various governments and their agencies as well as empowering and inculcating positive attitudes towards managing malaria effectively at home will help overcome these factors. Home management of malaria has proved to be the best strategy for effective treatment of uncomplicated malaria thereby preventing progression to severe illness and complications of malaria such as kidney failure, anemia, cerebral malaria etc. thereby reducing maternal and infant mortality and morbidity. There is therefore immense need to improve and sustain the home management of malaria strategy. This promises to be a veritable strategy in combating the disease especially in endemic resource poor settings.
